# Systematic literature review on the delays in the diagnosis and misdiagnosis of cluster headache

**DOI:** 10.1007/s10072-018-3598-5

**Published:** 2018-10-10

**Authors:** Alina Buture, Fayyaz Ahmed, Lisa Dikomitis, Jason W. Boland

**Affiliations:** 1 Department of Neurology, Hull Royal Infirmary, Hull, UK; 2 Hull York Medical School, University of Hull, Hull, UK; 3 School of Medicine and Research Institute Primary Care and Health Sciences, Keele University, Keele, UK; 4 Wolfson Palliative Care Research Centre, Hull York Medical School, University of Hull, Hull, UK

**Keywords:** Diagnostic error, Diagnostic mistake, Therapeutic error, Mismanagement, Unrecognised diagnosis

## Abstract

**Introduction:**

Patients with cluster headache (CH), the most common trigeminal autonomic cephalalgia, often face delayed diagnosis, misdiagnosis and mismanagement.

**Objectives:**

To identify, appraise and synthesise clinical studies on the delays in diagnosis and misdiagnosis of CH in order to determine its causes and help the management of this condition.

**Methods:**

The systematic review was prepared, conducted and reported in accordance with the Preferred Reporting Items for Systematic Review and Meta-Analysis. It was registered with International Prospective Register of Systematic Reviews. A systematic search of different electronic databases (Medline, EMBASE, PsycINFO, PubMed, CINAHL, BNI, HMIC, AMED, HBE and Cochrane Library) was carried out in May 2017. Reference lists of relevant articles were hand searched.

**Results:**

The search identified 201 unique studies. Fifteen studies met the inclusion criteria of which 13 case series studies and two survey studies. Nine studies assessed the delays in diagnosis and misdiagnosis of CH, five studies the delays in diagnosis and one study the misdiagnosis of CH. The studies included 4661 patients. Delays in diagnosis, misdiagnosis and mismanagement have been reported in many European countries, Japan and in the USA with well-developed health services. The patients with CH often visited many different clinicians, surgeons and dentists and received multiple diagnosis prior to being correctly diagnosed.

**Conclusion:**

This systematic review shows that the delays in the diagnosis of CH are a widespread problem, the time to diagnosis still vary from country to country and both patients and physicians are responsible for the delays in diagnosis.

## Background

Cluster headache (CH) is the most common of the trigeminal autonomic cephalalgias (TACs) and often described as the most severe pain possible [1]. The prevalence of CH is estimated at 0.5–3/1000, with male preponderance [2]. CH is characterised by attacks of unilateral pain associated with ipsilateral conjunctival injection, lacrimation, nasal congestion, rhinorrhoea, forehead and facial sweating, miosis, ptosis and/or eyelid oedema, and/or with restlessness or agitation [3, 4]. The CH attacks that can last between 15 min and 3 h occur from every other day to eight times a day [3]. Cluster headache is maximal orbitally, supraorbitally, temporally or in any combination of these sites, but may spread to other regions [3]. During the worst attacks, the intensity of pain is excruciating. Patients with CH, unlike those with migraine, are unable to lie down and characteristically pace and rock back and forth. The diagnosis of CH is based entirely on clinical history due to the lack of a diagnostic biomarker. Additionally, CH is uncommon and it is even rarer in the paediatric population, therefore underrecognised [5]. For these reasons, patients often face delays in diagnosis and misdiagnosis which inevitably leads to mismanagement. There have been no rigorous systematic literature reviews on this topic. The aim of this systematic literature review is to identify, appraise and synthesise all relevant clinical studies on the misdiagnosis and delays in the diagnosis of CH.

## Methods

The systematic review was prepared in accordance with the Preferred Reporting Items for Systematic Reviews and Meta-Analyses Protocols (PRISMA-P) 2015 guidelines [6] and was conducted and reported according to Preferred Reporting Items for Systematic Reviews and Meta-Analyses (PRISMA) [7]. It was registered with International Prospective Register of Systematic Reviews (PROSPERO) on 9/11/2017 (registration number CRD42017081204).

### Search strategy

A comprehensive search of different electronic databases was carried out in May 2017 to identify potential studies. The following electronic databases were searched: Medline, EMBASE, PsycINFO, PubMed, CINAHL, BNI, HMIC, AMED, HBE (NICE Healthcare Databases) and Cochrane Library. Pre-specified search criteria were designed with input from a professional librarian search specialist; Medical Subject Heading and free text terms were used to increase the search sensitivity.

To search for misdiagnosis, the search terms were misdiagnosis OR diagnostic error OR hidden diagnosis OR unrecognised diagnosis OR alternate diagnosis OR undiagnosed OR diagnostic mistake OR missed diagnosis. The search terms for delays in diagnosis were delays in diagnosis OR late diagnosis OR delayed diagnosis. These were combined with a search for cluster headache OR cluster-like headache. In addition to the electronic search, we screened the reference lists of the included articles and relevant literature known by the authors. The detailed search criteria are shown in Table 1.

**Table 1:** Databases and search criteria to identify articles on delays in diagnosis and misdiagnosis of CH

Database	Search term	Results
1. EMBASE	(((cluster ADJ5 headache*).ti,ab OR (cluster - like ADJ5 headache*).ti,ab OR exp “CLUSTER HEADACHE”/) AND ((misdiagnos*).ti,ab OR (diagnos* ADJ5 error*).ti,ab OR (hid* ADJ5 diagnos*).ti,ab OR (unrecognis* ADJ5 diagnos*).ti,ab OR (alternat* ADJ5 diagnos*).ti,ab OR (undiagnos*).ti,ab OR (diagnos* ADJ5 mistake*).ti,ab OR (miss* ADJ5 diagnos*).ti,ab OR exp “MEDICAL ERROR”/ OR exp “DIAGNOSTIC ERROR”/)) OR (((cluster ADJ5 headache*).ti,ab OR (cluster - like ADJ5 headache*).ti,ab OR exp “CLUSTER HEADACHE”/) AND ((delay* ADJ5 diagnos*).ti,ab OR (late ADJ5 diagnos*).ti,ab OR exp “DELAYED DIAGNOSIS”/))	138
2. PubMed	(((cluster ADJ5 headache*).ti,ab OR (cluster - like ADJ5 headache*).ti,ab) AND ((misdiagnos*).ti,ab OR (diagnos* ADJ5 error*).ti,ab OR (hid* ADJ5 diagnos*).ti,ab OR (unrecognis* ADJ5 diagnos*).ti,ab OR (alternat* ADJ5 diagnos*).ti,ab OR (undiagnos*).ti,ab OR (diagnos* ADJ5 mistake*).ti,ab OR (miss* ADJ5 diagnos*).ti,ab)) OR (((cluster ADJ5 headache*).ti,ab OR (cluster - like ADJ5 headache*).ti,ab) AND ((delay* ADJ5 diagnos*).ti,ab OR (late ADJ5 diagnos*).ti,ab))	104
3. Medline	(((cluster ADJ5 headache*).ti,ab OR (cluster - like ADJ5 headache*).ti,ab OR exp “CLUSTER HEADACHE”/) AND ((misdiagnos*).ti,ab OR (diagnos* ADJ5 error*).ti,ab OR (hid* ADJ5 diagnos*).ti,ab OR (unrecognis* ADJ5 diagnos*).ti,ab OR (alternat* ADJ5 diagnos*).ti,ab OR (undiagnos*).ti,ab OR (diagnos* ADJ5 mistake*).ti,ab OR (miss* ADJ5 diagnos*).ti,ab OR exp “MEDICAL ERRORS”/ OR exp “DIAGNOSTIC ERRORS”/)) OR (((cluster ADJ5 headache*).ti,ab OR (cluster - like ADJ5 headache*).ti,ab OR exp “CLUSTER HEADACHE”/) AND ((delay* ADJ5 diagnos*).ti,ab OR (late ADJ5 diagnos*).ti,ab OR exp “DELAYED DIAGNOSIS”/))	67
4. PsychINFO	(((cluster ADJ5 headache*).ti,ab OR (cluster - like ADJ5 headache*).ti,ab) AND ((misdiagnos*).ti,ab OR (diagnos* ADJ5 error*).ti,ab OR (hid* ADJ5 diagnos*).ti,ab OR (unrecognis* ADJ5 diagnos*).ti,ab OR (alternat* ADJ5 diagnos*).ti,ab OR (undiagnos*).ti,ab OR (diagnos* ADJ5 mistake*).ti,ab OR (miss* ADJ5 diagnos*).ti,ab)) OR (((cluster ADJ5 headache*).ti,ab OR (cluster - like ADJ5 headache*).ti,ab) AND ((delay* ADJ5 diagnos*).ti,ab OR (late ADJ5 diagnos*).ti,ab))	20
5. CINAHL	(((cluster ADJ5 headache*).ti,ab OR (cluster - like ADJ5 headache*).ti,ab OR exp “CLUSTER HEADACHE”/) AND ((misdiagnos*).ti,ab OR (diagnos* ADJ5 error*).ti,ab OR (hid* ADJ5 diagnos*).ti,ab OR (unrecognis* ADJ5 diagnos*).ti,ab OR (alternat* ADJ5 diagnos*).ti,ab OR (undiagnos*).ti,ab OR (diagnos* ADJ5 mistake*).ti,ab OR (miss* ADJ5 diagnos*).ti,ab OR (delay* ADJ5 diagnos*).ti,ab OR exp “DIAGNOSTIC ERRORS”/)) OR (((cluster ADJ5 headache*).ti,ab OR (cluster - like ADJ5 headache*).ti,ab OR exp “CLUSTER HEADACHE”/) AND ((delay* ADJ5 diagnos*).ti,ab OR (late ADJ5 diagnos*).ti,ab OR exp “DIAGNOSIS, DELAYED”/))	20
6. HBE	(((cluster ADJ5 headache*).ti,ab OR (cluster - like ADJ5 headache*).ti,ab OR exp “CLUSTER HEADACHE”/) AND ((misdiagnos*).ti,ab OR (diagnos* ADJ5 error*).ti,ab OR (hid* ADJ5 diagnos*).ti,ab OR (unrecognis* ADJ5 diagnos*).ti,ab OR (alternat* ADJ5 diagnos*).ti,ab OR (undiagnos*).ti,ab OR (diagnos* ADJ5 mistake*).ti,ab OR (miss* ADJ5 diagnos*).ti,ab OR exp “DIAGNOSTIC ERRORS”/)) OR (((cluster ADJ5 headache*).ti,ab OR (cluster - like ADJ5 headache*).ti,ab OR exp “CLUSTER HEADACHE”/) AND ((delay* ADJ5 diagnos*).ti,ab OR (late ADJ5 diagnos*).ti,ab))	1
7. BNI	(((cluster ADJ5 headache*).ti,ab OR (cluster - like ADJ5 headache*).ti,ab) AND ((misdiagnos*).ti,ab OR (diagnos* ADJ5 error*).ti,ab OR (hid* ADJ5 diagnos*).ti,ab OR (unrecognis* ADJ5 diagnos*).ti,ab OR (alternat* ADJ5 diagnos*).ti,ab OR (undiagnos*).ti,ab OR (diagnos* ADJ5 mistake*).ti,ab OR (miss* ADJ5 diagnos*).ti,ab)) OR (((cluster ADJ5 headache*).ti,ab OR (cluster - like ADJ5 headache*).ti,ab) AND ((delay* ADJ5 diagnos*).ti,ab OR (late ADJ5 diagnos*).ti,ab))	1
8. AMED	(((cluster ADJ5 headache*).ti,ab OR (cluster - like ADJ5 headache*).ti,ab) AND ((misdiagnos*).ti,ab OR (diagnos* ADJ5 error*).ti,ab OR (hid* ADJ5 diagnos*).ti,ab OR (unrecognis* ADJ5 diagnos*).ti,ab OR (alternat* ADJ5 diagnos*).ti,ab OR (undiagnos*).ti,ab OR (diagnos* ADJ5 mistake*).ti,ab OR (miss* ADJ5 diagnos*).ti,ab)) OR (((cluster ADJ5 headache*).ti,ab OR (cluster - like ADJ5 headache*).ti,ab) AND ((delay* ADJ5 diagnos*).ti,ab OR (late ADJ5 diagnos*).ti,ab))	0
9. HMIC	(((cluster ADJ5 headache*).ti,ab OR (cluster - like ADJ5 headache*).ti,ab) AND ((misdiagnos*).ti,ab OR (diagnos* ADJ5 error*).ti,ab OR (hid* ADJ5 diagnos*).ti,ab OR (unrecognis* ADJ5 diagnos*).ti,ab OR (alternat* ADJ5 diagnos*).ti,ab OR (undiagnos*).ti,ab OR (diagnos* ADJ5 mistake*).ti,ab OR (miss* ADJ5 diagnos*).ti,ab)) OR (((cluster ADJ5 headache*).ti,ab OR (cluster - like ADJ5 headache*).ti,ab) AND ((delay* ADJ5 diagnos*).ti,ab OR (late ADJ5 diagnos*).ti,ab))	0
10. Cochrane Library	#1 cluster near/5 headache*:ti,ab,kw (Word variations have been searched) #2 cluster-like headache*:ti,ab,kw (Word variations have been searched #3 MeSH descriptor: (Cluster headache) explode all trees #4 misdiagnos* #5 diagnos* near/5 error* #6 hid* near/5 diagnos* #7 unrecognis* near/5 diagnos* #8 alternat* near/5 diagnos* #9 undiagnos* #10 diagnos* near/5 mistake* #11 miss* near/5 diagnos* #12 MeSH descriptor: (Diagnostic error) explode all trees #13 delay* near/5 diagnos* #14 late near/5 diagnos* #15 MeSH descriptor (Delayed diagnosis) explode all trees #16 {or #1-#3} #17 {or #4-#12} #18 {or #13-#15} #19 {and #16-#17} #20 {and #16, #18} #21 {or #19-#20}	1
Total number of references		352
Deduplicates removed		154
Total		198

Two authors (AB and JB) independently assessed all titles and abstracts for inclusion. The inclusion/exclusion criteria implemented for all searches are shown in Table 2. Full-text papers were retrieved for those meeting the inclusion criteria and for those articles whose eligibility criteria could not be assessed based only on the title and abstract. Two authors (AB and JB) independently assessed all full-text articles and disagreement was resolved by discussion to reach consensus and if needed with the intervention of a third reviewer (FA). The findings are reported according to PRISMA guidelines [7].

**Table 2:** The inclusion and exclusion criteria

Inclusion	Exclusion
Study design	
Prospective and retrospective studies, case series and survey studies on misdiagnosis and/or delays in the diagnosis of CH	Case reports
Participants	
Children or adult patients with a diagnosis of CH according to ICHD criteria confirmed by a neurologist	Children or adult patients with a diagnosis of CH not based on ICHD criteria and not confirmed by a neurologist, studies with less than 10 participants
Date	
There will be no restrictions by date	
Geographical location	
There will be no restrictions by geographical location	
Language	
There will be no restrictions by language. Non-English language articles will be included and all the foreign language articles will be translated. However, if the translation is not possible, it will be recorded	

### Data extraction, assessment and analysis

The data was independently extracted by two authors (AB and JB). Data extracted included the study design, methods of data acquisition, study population (number of participants, men:women ratio, percentage of patients with episodic cluster headache (ECH) and chronic cluster headache (CCH)), time from disease onset to diagnosis (the patient’s delay: the mean time between the CH attack and first consultation of a clinician, clinician’s delay: the mean time between the first consultation of a clinician and correct diagnosis and the mean total delay: sum of patient’s delay and clinician’s delay), percentage of patients misdiagnosed, diagnosis received prior to CH diagnosis, the type and number of clinicians seen prior to diagnosis, treatment received prior to diagnosis and factors involved in the diagnostic delay. The discrepancies were resolved through discussion with a third reviewer (FA).

### Risk of bias in individual studies

The risk of bias in individual studies was conducted in order to assess the quality of the studies included in the SLR. Quality assessment was performed using the Joanna Briggs Institute (JBI) Appraisal Checklist for case series studies [8]. Ten domains of the study design and reporting were assessed, each rated ‘Yes’, ‘No’, ‘Unclear’ or ‘Not applicable’. The Oxford Centre for Evidence-Based Medicine (OCEBM) critical appraisal was used for survey studies [9]. Ten domains of the study design and reporting were assessed, each rated ‘Yes’, ‘No’, ‘Unclear’ or ‘Not applicable’. Studies were not excluded based on their quality appraisal. The studies were independently assessed by two reviewers (AB and JB) and the discrepancies were resolved through discussion with a third author (FA).

#### Data Availability

All data is fully available without restriction.

## Results

### Studies included

**Fig. 1:**
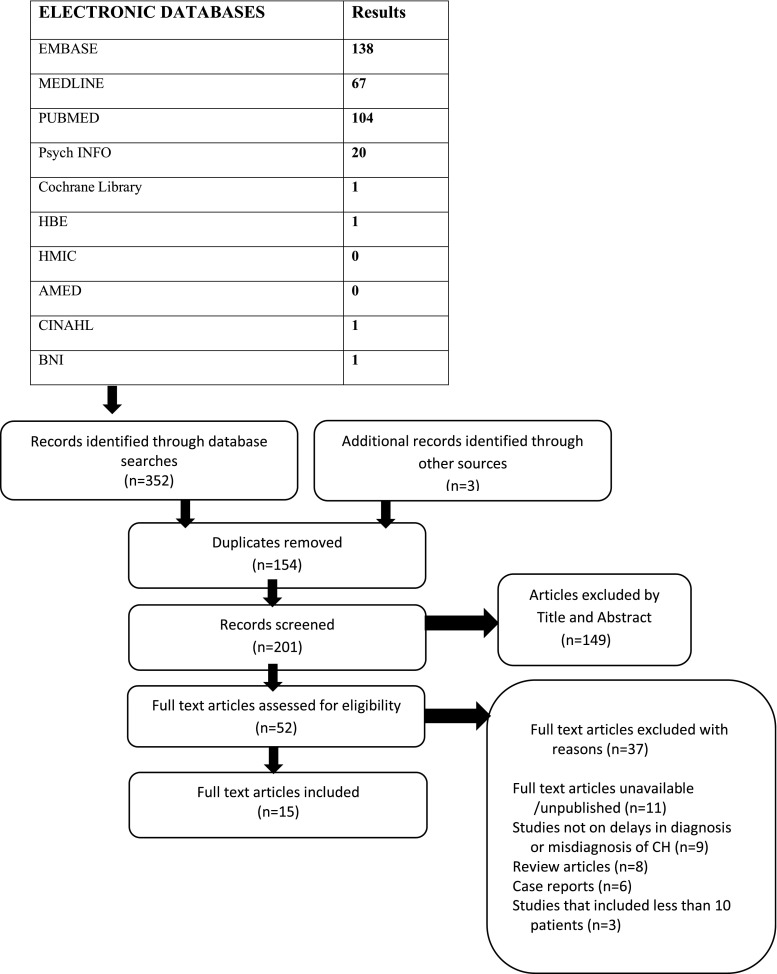
PRISMA flow diagram of study selection based on Preferred Reporting Items for Systematic Review and Meta-Analysis Protocols

The search carried out in May 2017 on diagnostic delays and misdiagnosis of CH identified 201 unique studies (Fig. 1). The retrieved articles were published between January 1978 and May 2017. All studies were screened by title and abstract and 149 articles were excluded at this stage. Full-text articles were assessed for the remaining 52 studies and 15 studies met our inclusion criteria (Table 2). Thirty-seven articles were excluded after the full-text screening; the reasons for exclusion are shown in the PRISMA flow chart (Fig. 1). The 15 included studies took place in Europe, the USA and Asia. Four studies were from the USA, 3 from Denmark and 1 each from Greece, Serbia, Spain, Norway, Japan, Britain and Flanders. One study was conducted in multiple countries: Italy, Moldova, Ukraine and Bulgaria.

Thirteen case series studies and two survey studies were included. Nine studies assessed the delays in diagnosis and misdiagnosis of CH, five studies the delays in diagnosis and one study the misdiagnosis of CH. The studies included a total of 4661 patients, aged 3–81 years, men and women with ECH and CCH. The percentage of patients with ECH varies from 64 to 100%. The male to female ratio varied from 1.9:1 [10] to 9.6:1 [11]. One included study was in children with CH [12]. The data extracted from case series and survey studies is shown in Table 3 and Table 4. The values in Tables 3 and 4 are extracted from the original (referenced) papers and the percentage values are rounded to the nearest integer. The number of patients with ECH and CCH was converted into percentages where necessary for consistency. The ratio (men:women) was calculated if it was not provided in the cited work.

**Table 3:** Data extracted from case series and survey studies

Country	Authors	Number of patients and men:women ratio (*R*)	Study design	Methods of data acquisition	ECH and CCH (%)	Time from disease onset to diagnosis (years) and the *p* value		Misdiagnosis and percentage of patients misdiagnosed (%)	Type and mean number of clinicians seen prior to diagnosis	Treatment received prior to diagnosis
Denmark	Lund et al. (2017)	351 *R* = 2:1	Retrospective study	362-item questionnaire and structured interview	64 ECH 36 CCH	Mean total delay 6.2 total group 6.56 men 5.50 women	*p* = 0.21	Migraine 25% Tension-type headache 19% Sinusitis 14% 61% women and 46% men misdiagnosed	NR	NR
Greece	Vikelis and Rapoport (2016)	302 *R* = 3.6:1	Retrospective study	Semi-structured questionnaire and neurological examination	78 ECH 22 CCH	Median total delay (range) < 1989 20 (0–45) 18 (0–41) men 23 (20–45) women 18 (0–45) ECH 30 (20–30) CCH 1990–1999 12 yrs (2–21) 12 (3–21) men 12 (2–16) women 11 (2–21) ECH 13 (2–16) CCH 2000–2009 5 (0–14) 5 (0–12) men 3 (0–14) women 5 (0–14) ECH 5 (0–12) CCH 2010–2015 1 (0–7) 0 (0–6) men 3 (0–7) women 1 (0–7) ECH 1 (0–6) CCH		Migraine 51% Trigeminal neuralgia 42% Ophthalmic disease 11% Dental or jaw disease 15% ENT disease 25% Cervical spine disease 12%	Primary care physician 65% Dentist 26% ENT specialist 36% Ophthalmologist 31% Neurologist 41% Neurosurgeon 9% Other 23% Self-diagnosis 13%	Pharmaceutical treatment 63% Unnecessary procedures 14% Dentists 10% ENT 10%
							*p* = 0.01			
Serbia	Zidverc-Trajcovic et al. (2014)	182 *R* = 1.9:1	Retrospective case series	Clinical note review	89 CH 11 CCH	Mean total delay 7.8 ± 8.0 (whole group) < 20 yrs age of onset 13.8 ± 9.7 20–40 yrs age of onset 7.9 ± 7.6 > 40 yrs age of onset 4.2 ± 2.1 69% of patients had a diagnostic delay longer than 2 yrs	*p* = 0.000	NR	NR	NR
Italy Moldova Ukraine Bulgaria	Voiticovski-Iosob et al. (2014)	144 *R* = 2.7:1	Consecutive case series	Clinical examination (74%) and 20-item questionnaire delivered over the phone (26%)	100 ECH	Mean total delay 5.3 ± 6.4 (range 0–30) Eastern European countries: 4.0 ± 3.7 Italy: 5.6 ± 6.9 Patient delay 24% (did not seek medical advice)	NR	Trigeminal neuralgia 29% Migraine without aura 23% Sinusitis 17% Headache attributed to idiopathic intracranial hypertension 6% Tension-type headache 6% Dental problems 4% Depression 4% Questionable CH 3% Self-diagnosis 15% 77% patients misdiagnosed	Neurologists 49% General practitioners 35% ENT specialists 10% Dentist 3% Other 4% (ophthalmologist, paediatrician, anaesthesiologist, cardiologist, emergency medicine) 2.6 clinicians/patient	131/144 symptomatic treatment 91% (of these: triptans 17%, oxygen 1%, NSAIDS 55%, combination of analgesics 18%) 33/144 preventative medication 23% 44/144 non-pharmacological treatment 31% (of these: acupuncture 32%; physical therapy 16%; relaxation techniques 11%; cold therapy 9%; tooth extraction 16%; sinus medications aerosol 2%; other drugs, cannabis, marijuana, alcohol 9%; homoeopathy; chirotherapy 5%)
Spain	Sanchez del Rio et al. (2014)	75 *R* = 8.3:1	Consecutive case series	10-item questionnaire study	NR	Mean total delay 4.9 (range 1–28 mts)	NR	Migraine 45% No diagnosis 28% Trigeminal neuralgia 25% Sinusitis 19% Dental pain/jaw disease 16% Psychiatric 9% SUNCT 3% 57% patients misdiagnosed (28% no specific diagnosis)	4.6 clinicians/ patient (range 1–12)	No information or inappropriate treatment 60%
Norway	Bekkelund et al. (2014)	70 *R*: 4.8:1	Patients identified in the registers of two neurological departments	Questionnaire and diagnosis confirmed through clinical chart or over the phone	NR	Median total delay 4 (range 0–30)	NR	NR	NR	Acupuncture 29% Chirotherapy 19% Physiotherapy 1% Cannabis 1% Naprapathic treatment 1% Healing 1% Scuba diving 1% Reflexology 1% Dental treatment 1%
USA	Rozen and Fishman (2012)	1134 *R* = 3.8:1	Nationwide survey study	187-item questionnaire (website based)	NR	Total delay percentage: < 1 (25%) 1 yr (7%) 2 yrs (10%) 3 yrs (9%) 4 yrs (6%) 5 yrs (7%) 6 yrs (4%) 7 yrs (4%) 8 yrs (4%) 9 yrs (2%) 10+ (22%) > 5 yrs in 42% patients	NR	Migraine 34% Sinusitis 21% Allergies 6% Tooth-related issues 5%	NR	NR
Japan	Imai et al. (2010)	86 *R* = 3.8:1	Consecutive case series	Structured interview	96 ECH 4 CCH	Mean total delay 7.3 ± 6.9 yrs (range 0–28)	NR	NR	NR	NR
Flanders	Van Alboom et al. (2009)	85 *R* = 9.6:1	Consecutive case series	Self-administered 90-item questionnaire	79 ECH 21 CCH	Mean total delay 44 mts Physician’s delay Mean 35 mts Patient’s delay Mean 11 mts < 1 yr (54%) 2–4 yrs (14%) 5–10 yrs (18%) 10+ yrs (13%)	NR	Migraine 45% Sinusitis 23% Tooth/jaw problem 23% Tension-type headache 16% Trigeminal neuralgia 16% Ocular problem 10% Neck/back problem 7% Nasal problem 5% 65% patients misdiagnosed	NR	Non-specific analgesia (79%) 46/85 invasive therapy (of these: dental procedures 21%; sinus surgery 10%) Inappropriate preventative treatments (carbamazepine 12%; propranolol 12%; amitriptyline 9%) 40/85 alternative therapies 47% (of these: acupuncture 26%; osteopathy 18%; chiropractics 15%; homoeopathy 13%; herbal therapy 11%; spiritual healing 7%; reflexology 6%; hypnosis 2%)
Denmark	Jensen (2007)	85 *R*: 1.9:1	Case series study	Semi-structured 97-question telephone interview and clinical note review	79 ECH 20 CCH 1 undetermined	Mean total delay 9 (range 0–39) whole group ECH: 8 (range 0–35) CCH 9 (range 0–39)	NR	NR	44.7% (38/85) of patients had previously been admitted to hospital due to CH	Non-medical treatment was received by 58% (49/85)
UK	Bahra and Goadsby (2004)	230 *R*: 2.5:1	Case series study (24%) and patients recruited from national support groups (76%)	Interview and questionnaire (telephone or face-face)	79 ECH 21 CCH	Mean total delay Before 1950 12 yrs 1950–1959 22.3 yrs 1960–1969 17.2 yrs 1970–1979 9.5 yrs 1980–1989 6.4 yrs 1990–1999 2.6 yrs	NR	NR	Dentist 45% ENT specialist 27% Optician 32% Ophthalmologist 15% Other (physician, migraine clinic, neurosurgeon, psychiatrist, pain clinic) 7% Self-diagnosis 13%	Tooth extraction, splint, brace, filling, X-rays, maxillo-facial surgery 18% Sinus washout, surgery for nasal septum deviation, antibiotics, X-rays 13% Spectacle prescription altered, eye-exercises 3%
Denmark	Van Vliet et al. (2003)	1163 *R*: 3.7:1	National mailing via headache groups and to Dutch general practitioners and neurologists invited them to refer patients with a possible diagnosis of CH	Questionnaire	73 ECH 21 CCH 6 undetermined	Median total delay 3 yrs (range 1 week and 48 yrs)	NR	Sinusitis 21% Migraine 17% Dental-related pain 11%	Dentists 34% ENT specialists 33% Alternative therapists 33%	Tooth extraction 16% ENT operation 12%
USA	Klapper et al. (2000)	686	Patients accessing CH website were invited to participate in an internet survey	28-item questionnaire	85 ECH 15 CCH	Mean total delay 6.6 yrs	NR	3.9 (average number of incorrect diagnoses)	4.3 clinicians/ patient (average)	NR
USA	Maytal et al. (1992)	35 *R*: 6:1	Case series study	Semi-structured interviews	86 ECH 14 CCH	Mean total delay 8.5 (range 0–34) 8.5 (range 0–34)	NR	NR	Neurologists or headache specialists 71% Internists or general practitioners 37% Otolaryngologists 26% Paediatricians 26% Ophthalmologists 23% Psychiatrists 11% Chiropractors 6% Orthopaedic surgeons 3% Allergists 3%	Surgical repair of a deviated septum (1)
USA	Bittar and Graff-Radford (1992)	33 *R*: 3:1	Retrospective consecutive case series	Clinical note review	NR	NR	NR	NR	NR	Headache compounds (Fiorinal, Fioricet, Cafergot, Midrin) NSAIDS (Aspirin, Dolobid, Motrin) Membrane stabilising drugs (Tegretol, Dilantin, Lioresal) Narcotics (Dilaudid, codeine, MS Contin) Tricyclic antidepressants Dental procedures (oral orthosis 18%; teeth extracted 12%; coronoplasty 9%; root canal treatments 6%)

*R*, men:women ratio; *ECH*, episodic cluster headache; *CCH*, chronic cluster headache; *p*, *p* value; yrs, years; mts, months; ENT, ears, nose and throat

**Table 4:** Factors involved in the diagnostic delay (data available from 3 studies)

Country	Greece			Denmark			Denmark
Author	Vikelis and Rapoport (2006)			Van Vliet et al. (2003)			Van Alboom et al. (2009)
Factors involved in the diagnostic delay		Years to diagnosis Median (range)	*p* value	% of patients with clinical features	Years to diagnosis Median (range)	*p* value	Lower age at onset
	Decade of onset		0.001	Male gender (79%)		0.448	Pain that does not reach the peak within the first 5 min
	< 2000	13 (0–45)		Yes	3 (< 1–45)		*p* < 0.05
	2000–2009	5 (0–14)		No	3 (< 1–48)		
	≥ 2010	1 (0–7)		Episodic CH (73%)		0.001	
	Side shift between bouts		0.008	Yes	3 (< 1–48)		
	No	5 (0–45)		No	1 (< 1–28)		
	Yes	8 (0–26)		Nausea during attacks (27%)		0.001	
	Jaw location of pain		0.002	Yes	4 (< 1–45)		
	No	5 (0–30)		No	2.3 (< 1–48		
	Yes	7 (0–45)		Vomiting during attacks (12%)		0.003	
	Cheek location of pain		0.015	Yes	4.8 (< 1–37)		
	No	5 (0–30)		No	2.5 (< 1–48)		
	Yes	7 (0–45)		Photophobia/phonophobia (54%)		0.022	
	Lower teeth location of pain		0.015	Yes	3 (< 1–48)		
	No	5 (0–30)		No	2 (< 1–48)		
	Yes	10 (0–45)		Nocturnal onset of attacks (78%)		0.009	
	Ear location of pain		0.041	Yes	3 (< 1–48)		
	No	5 (0–41)		No	2 (< 1–35)		
	Yes	10 (0–45)		Interictal headache (16%)		0.078	
	Photophobia		0.016	Yes	2 (< 1–42)		
	No	4 (0–30)		No	3 (< 1–48)		
	Yes	6 (0–45)		Circadian rhythm (64%)		0.459	
	Aggravation by physical activity		0.008	Yes	3 (< 1–48)		
	No	3 (0–20)		No	2.5 (< 1–40)		
	Yes	6 (0–45)		Restlessness (76%)		0.787	
	Forehead and facial sweating		0.018	Yes	3 (< 1–48)		
	No	5 (0–30)		No	2 (< 1–37)		
	Yes	6 (0–45)		Pain radiating to jaw (37%)		0.387	
	Absence of autonomic features		0.023	Yes	3 (< 1–42)		
	No	2 (0–14)		No	2.5 (< 1–48)		
	Yes	5 (0–45)		Alternating attack side (11%)		0.001	
				Yes	6 (< 1–34)		
				No	2.5 (< 1–48)		

### Non-English articles

Four full-text articles in foreign languages were identified and translated [13–16]. The articles were excluded as they did not meet the inclusion criteria (the studies were not on delays in diagnosis or misdiagnosis of CH).

### Risk of bias in individual studies

The 13 case series assessed using JBI Appraisal Checklist (Table 5) were consecutive case series [11, 12, 17–20] and non-consecutive case series [21–23] which scored ‘YES’ to all JBI domains as well as retrospective case series [10, 24] and one study with unclear inclusion of participants [25]. The two survey studies were assessed using OCEBM critical appraisal of a survey (Table 6). Using this tool, we identified studies that did not assess the statistical significance [26, 27] and did not give the confidence intervals for the main results [27]. We did not exclude studies based on their quality appraisal.

**Table 5:** The Joanna Briggs Institute (JBI) critical appraisal tool for case series

Author	Were there clear criteria for inclusion?	Was the condition measured in a standard, reliable way for all participants?	Were valid methods used for identification of the condition for all participants included?	Did the case series have consecutive inclusion of participant s?	Did the case series have complete inclusion of participants?	Was there clear reporting in the demographic of the participants?	Were there clear reporting of clinical information of the participants?	Were the outcomes or follow-up results of cases clearly reported?	Was there clear reporting in the presenting site(s)/clinic(s) demographic information?	Was statistical analysis appropriate?
Lund et al. (2017)	Yes	Yes	Yes	No	No	Yes	Yes	Yes	Yes	Yes
Vikelis and Rapoport (2016)	Yes	Yes	Yes	Yes	Yes	Yes	Yes	Yes	Yes	Yes
Zidverc-Traj covic et al. (2014)	Yes	Yes	Yes	No	No	Yes	Yes	Yes	Yes	Yes
Voiticovski-Iosob et al. (2014)	Yes	Yes	Yes	Yes	Yes	Yes	Yes	Yes	Yes	Yes
Sanchez del Rio et a1. (2014)	Yes	Yes	Yes	Yes	Yes	Yes	Yes	Yes	Yes	Yes
Bekkelund et al. (2014)	Yes	Yes	Yes	Yes	Yes	Yes	Yes	Yes	Yes	Yes
Imai et al. (2010)	Yes	Yes	Yes	Yes	Yes	Yes	Yes	Yes	Yes	Yes
Van Alboom et al. (2009)	Yes	Yes	Yes	Yes	Yes	Yes	Yes	Yes	Yes	Yes
Jensen (2007)	Yes	Yes	Yes	Yes	Yes	Yes	Yes	Yes	Yes	Yes
Bahra and Goadsby (2004)	Yes	Yes	Yes	No	No	Yes	Yes	Yes	Yes	Yes
Van Vliet et al. (2003)	Yes	Yes	Yes	No	No	Yes	Yes	Yes	Yes	Yes
Maytal et al. (1992)	Yes	Yes	Yes	Yes	Yes	Yes	Yes	Yes	Yes	Yes
Bittar-Graff Radford (1992)	Yes	Yes	Yes	Unclear	Unclear	Yes	Yes	Yes	Yes	Yes

**Table 6:** Oxford Centre for Evidence-Based Medicine (OCEBM) critical appraisal of survey studies

Author	Did the study address a clearly focused question/issue?	Is the study design appropriate for answering the research question?	Is the method of selection of subjects clearly described?	Could the way the sample was obtained introduce selection bias?	Was the sample of subjects representative with regard to the population to which the findings will be referred?	Was the sample size based on pre-study consideration of statistical power?	Was a satisfactory response rate achieved?	Are the measurements likely to be valid and reliable?	Was the statistical significance assessed?	Are the confidence intervals given for the main results?	Could there be confounding factors that haven’t been accounted for?	Can the results be applied to your organisation?
Rozen and Fisherman (2012)	Yes	Yes	Yes	No	Yes	No	Yes	Yes	No	No	No	Yes
Klapper et al. (2000)	Yes	Yes	Yes	No	Yes	No	Yes	Yes	No	Yes	No	Yes

### Diagnostic delays

Fourteen of the 15 studies investigated the total delay in diagnosis (i.e. the time from disease onset to correct diagnosis). The studies reported different statistics for time to correct diagnosis (mean, median or percentage). Ten studies assessed the mean time to correct diagnosis [10–12, 18–21, 23, 26, 28], three studies the median time [17, 22, 24] and one study the percentage of patients that experienced delays in diagnosis [29]. The mean time to correct diagnosis recorded in the UK was 2.6 years (between 1990 and 1999) [21], in Flanders 3.6 years [11], in Spain 4.9 years [18], in Italy and East European countries 5.3 ± 6.4 years [28], in Denmark between 6.2 years [23] and 9 years [20], in the USA between 6.6 [26] and 8.5 years [12], in Japan 7.3 ± 6.9 years [19] and in Serbia 7.8 ± 8 years (quoted verbatim form the original paper) [10]. The median time to correct diagnosis was 1 year (range 0–7) in Greece [17], 3 years (range 1–48) in Denmark [22] and 4 years (range 0–30) in Norway [24]. In one study performed in the USA, 42% of patients waited more than 5 years to receive a correct diagnosis of cluster headache [29].

Two studies showed a reduction in delay in the diagnosis of CH over time, from 22.3 years (before 1959) to 2.6 years (between 1990 and 1999) in the UK [21] and from 20 years (prior to 1989) to 1 year (between 2010 and 2015) in Greece [17]. Two studies looked at patient’s and clinician’s delays in the diagnosis of CH [11, 28]. Van Alboom et al. showed that the mean time between the first cluster headache attack and the first consultation was 11 months [11] and Voiticovski-Iosob et al. found patient’s delay in almost one quarter of cases [28].

While Bahra and Goadsby found no significant difference in time to diagnosis between men and women [21], Lund et al. showed that men waited a mean time of 6.56 years and women waited 5.5 years [23]. Gender difference was also recorded by Vikelis and Rapoport where a median of 0 years (range 0–6) was found for men and 3 years (range 0–7) for women [17]. One study assessed the influence of age of onset on the diagnostic delay [10]. Zidverc-Trajkovic et al. showed that the condition is less recognised in patients with early onset of CH (less than 20 years of age) [10]. People with late onset of CH (> 40 years of age) were more rapidly diagnosed than subjects with typical age of onset of CH (20–40 years of age) [10]. In the study conducted by Van Vliet et al., the patients with ECH had longer delays in diagnosis compared to CCH patients [22], probably due to longer remission periods.

### Misdiagnoses prior to correct CH diagnosis

Migraine, trigeminal neuralgia, sinusitis and dental/jaw disease are the most common misdiagnoses. Other diagnoses received by the CH patients were tension-type headache; ophthalmic disease; ear, nose and throat (ENT) disease; cervical spine disease; idiopathic intracranial hypertension; allergies; short lasting neuralgiform headache with conjunctival injection and tearing (SUNCT) and psychiatric disorders. Migraine was the most received misdiagnosis [11, 17, 18] followed by trigeminal neuralgia, [17, 18, 28]. Sinusitis was often diagnosed in patients with CH, most likely due to presence of rhinorrhoea, nasal congestion and seasonal variation, although there was no significant statistical correlation between these features and the diagnosis of CH [11]. The mean number of diagnosis received per patient was 2.2 in Italy and Eastern Europe [28] and 3.9 in the USA [26]. In Flanders, 65% of the patients studied were misdiagnosed [11] and in Italy and East Europe 77% were misdiagnosed [28]. In Denmark, more women (61%) were misdiagnosed as migraine compared to men (45.5%) [23].

### Clinicians seen prior to correct CH diagnosis

Patients with CH were often seen by different clinicians before the correct diagnosis was made. Vikelis and Rapoport showed that nearly two thirds of their Greek patients (63.5%) consulted a general practitioner or internist, around one third an ENT specialist, ophthalmologist or dentist, and a small proportion (8.5%) a neurosurgeon [17]. In the same study, 40% of the patients were seen by neurologists who missed the diagnosis [17]. In Flanders, neurologists correctly diagnosed 80% of cases [11]. Patients often sought help from alternative medicine specialists (acupuncturists and chiropractors) [11, 24, 25, 28]. Even children consulted many different specialists prior to diagnosis (internists, general practitioners, otolaryngologists, opthalmologists, psychiatrists, chiropractors, orthopaedic surgeons and allergists) [12]. Self-diagnosis using different sources of information (internet, reading about CH and discussion with other people suffering with CH) with subsequent medical confirmation was the second most common way of diagnosis after clinician’s diagnosis [17] and it was reported in 4%, 13% and 15% of patients in Flanders [11], the UK [21] and Italy and East European countries respectively [28]. Patients consulted between 2 and 5 clinicians before the correct diagnosis was made [11, 17, 18, 28] frequently including a dentist, ENT specialists or ophthalmologist who exceptionally made the diagnosis [11]. Vikelis and Rapoport found that patients with CCH consulted more clinicians than patients with ECH (median 4 vs 2) [17] and no differences in the number of clinicians consulted by men and women were found [17]. Most patients with CH have never been seen by specialists in emergency medicine [29]. The most obvious explanation would be the short duration of the attacks.

### Mismanagement prior to correct CH diagnosis

General neurologists frequently offered non-evidence-based CH treatments [12, 17, 28]. Dentists and ENT specialists performed tooth extractions, fillings, sinus washout and surgery for nasal septum deviation without any success. Dentists, ENT specialists or other clinicians that did not recognise the disorder often recommend unnecessary investigations (MRI head, CT head, EEC, cervical spine X-ray, skull X-ray) to diagnose a secondary headache [28]. Patients underwent alternative medicine treatments such as acupuncture [11, 24, 25, 27], homoeotherapy [28], chirotherapy [24, 25, 28], relaxation techniques [28], cold therapy [28], reflexology [11], hypnosis [11], osteopathy [11], spiritual healing [11] and illicit drug use [24, 28]. Even after correct diagnosis of CH, the patients complained of lack of information regarding the cause of the disorder and available treatments [18]. Some patients received incorrect information as to the cause of CH (psychiatric, vascular disorder, genetic/familial, brain injury, alcohol, tobacco) and others no information [18].

### Factors involved in the diagnostic delay and misdiagnosis

Three studies assessed the factors involved in the diagnostic delay [11, 17, 22]. Van Vliet et al. showed that the presence of ECH, nausea, vomiting during attacks, photophobia or phonophobia, nocturnal onset of attacks, restlessness, pain radiating to the jaw, alternating attack side and circadian rhythm delayed the diagnosis of CH [22]. The male gender and interictal headache did not influence the correct diagnosis of CH [22]. However, Vikelis and Rapoport showed that the side shift between bouts, jaw location of pain, the cheek location of pain, lower teeth location of pain, ear location of pain, aggravation by physical activity, the presence of forehead and facial sweating, the presence of photophobia and the absence of cranial autonomic features delayed the correct diagnosis of CH [17]. The authors have also shown that the decade of onset of CH influenced the correct diagnosis [17]. Patients with onset before the year 2000 waited a median of 13 years (range 0–45) to be diagnosed compared to patients with onset after the year 2010 who waited a median of 1 year (range 1–7) [17]. A lower age of onset and pain that does not reach the maximum intensity within the first 5 min were also features that contributed to diagnostic delay [11].

## Discussion

It is evident from the studies that diagnostic delay in CH is not confined to a geographical area. Although some countries had less delay than others, delays in diagnosis were recorded in multiple countries in Europe, the USA and Japan. One possible reason could be limited knowledge about the characteristics of CH across countries. However, these results should be interpreted with caution as each study does not reflect the whole CH population of a country. Only one nationwide survey study performed in the USA that included a sample of 1134 patients was retrieved by our searches and could be considered representative for a large cohort of patients with CH [29]. The studies were performed over a period of 25 years and are not directly comparable as the International Classification of Headache Disorders has suffered amendments over the years.

The studies included in this review showed that patient’s delay in diagnosis is as important as clinician’s delay [11, 28]. The reason why patients with CH do not seek timely medical advice is not well understood. The short duration of the attacks could be an explanation although there are currently no studies that assessed this.

It has been shown that the episodic pattern of attacks, a specific feature of CH, does not seem to contribute to an earlier diagnosis [22]. Moreover, extended periods of remissions only prolong the diagnostic delay. Improved awareness of the condition is the most probable reason for the reduction of time to correct diagnosis in the UK, Greece and Denmark [17, 20, 21, 23]. It is unclear why patients with late onset CH were more rapidly diagnosed than those with early onset [10]. It is possible that clinicians erroneously view CH as a disorder with onset predominantly in late adulthood. Another explanation might be that clinicians are more suspicious of a sinister cause for the symptoms if the patient is older, and therefore have a lower threshold to refer to a neurologist although there are no studies that have assessed this.

A lack of knowledge of the characteristics of CH is likely to influence the clinician to seek an alternative diagnosis. Some CH characteristics could lead the clinician astray. For example, migraine features (e.g. aura, photophobia, phonophobia, nausea, vomiting) and a family history of migraine are often encountered in patients with CH [22]. The features of the pain in CH may also mislead the clinician in making the wrong diagnosis. Although CH affects the first division of the trigeminal nerve while trigeminal neuralgia the second or third and exceptionally the first division, trigeminal neuralgia was the second most received misdiagnosis in two studies [17, 18]. The presence of stereotyped attacks associated with cranial autonomic symptoms, the absence of triggers and the totally different duration and pain quality still qualify trigeminal neuralgia as one of the most received misdiagnosis [17, 18, 28]. It is possible that clinicians are more aware of trigeminal neuralgia, even though CH is more common (incidence 53/100.000 [30] vs 4.5/100.00 [31]) but there are no studies that validated this. The presence of side shift between attacks was also correlated with diagnostic delay possibly because CH is defined as ‘unilateral pain’ as per ICHD-3 criteria [3].

Misdiagnosis invariably leads to mismanagement. In CH, due to the severity of the symptoms, patients desperately seek the opinion of several specialists until the symptoms are alleviated. It is possible that some specialists feel the need to offer invasive procedures in an attempt to provide some form of relief, even if the chance of success is small. A high proportion of patients with CH undergo invasive procedures from dental surgeons and ENT specialists when a clear indication for such interventions was lacking. These results suggest that further awareness is required, particularly in the dental and ENT professions regarding the pain and cranial autonomic symptoms of CH mimicking dental and sinus pathologies, to avoid unnecessary and potentially harmful procedures.

In an attempt to treat their symptoms, patients with CH are more likely to employ extreme measures. The use of illicit drugs among CH sufferers is common [24, 28]. They are also more inclined to have recourse to non-evidence-based and non-pharmocological treatments [11, 24]. This further supports the need for timely diagnosis and initiation of evidence-based treatments, and patient education. The evidence suggests that even after the correct diagnosis is reached, some patients received poor or incorrect information about the nature of their disability [18]. Suboptimal management is not limited to the cluster headache sufferers since most headache patients are undertreated, hence the importance of headache centres and promoting education of GPs [32].

### Strengths

This is the first rigorously conducted systematic review on delays in diagnosis and misdiagnosis of cluster headache. A detailed search strategy of 10 electronic databases was used with no date or language restrictions. We included larger studies that could demonstrate rigorous analysis and we have excluded studies with less than 10 patients and case reports.

### Limitations

It is possible that relevant studies were missed despite a comprehensive search strategy across multiple databases with no date or language restrictions. Due to the paucity of studies in this area, we did not exclude studies on the basis of quality appraisal.

### Future work

As CH is a life-long severe and debilitating condition that requires prompt diagnosis and management, it is essential to establish what factors are involved in the diagnostic delay and misdiagnosis. Educational activities for general practitioners, ENT specialists, ophthalmologists and other medical specialities and even for neurologists are important to raise awareness of CH, its diagnosis and management. Getting medical and emotional support are important priorities for CH sufferers. Clinicians of all specialities should be aware of the existence of CH and long-term support should be in place so that patients with CH can live a normal life. Future work regarding biomarkers could help in the misdiagnosis and delays in the diagnosis of CH.

## Conclusions

Delays in diagnosis, misdiagnosis and mismanagement of CH are a widespread problem and have been reported in many countries with well-developed health services, including several European countries, Japan and in the USA. Both patient and clinician factors account for the delays in diagnosis. Patients with CH often waited before seeking medical advice and when they did, they visited many clinicians and received multiple misdiagnosis prior to being correctly diagnosed. The failure to diagnose patients with CH leads to poor management, disability and misuse of healthcare resources. If a clinician has a suspicion of CH, this should trigger referral to specialised headaches centres for a correct diagnosis and initiation of appropriate treatment and to minimise the wastage of healthcare resources and unnecessary procedures.

## Abbreviations

CH: cluster headache
PRISMA: Preferred Reporting Items for Systematic Review and Meta-Analysis
PROSPERO: International Prospective Register of Systematic Reviews
TACs: trigeminal autonomic cephalalgias
ECH: episodic cluster headache
CCH: chronic cluster headache
OCEBM: Oxford Centre for Evidence-Based Medicine
JBI: Joanna Briggs Institute
ENT: ear, nose and throat
SUNCT: short lasting neuralgiform headache with conjunctival injection and tearing
